# Evaluation of the Effectiveness of Liraglutide on Metabolic Parameters in the Treatment of Obesity

**DOI:** 10.7759/cureus.50544

**Published:** 2023-12-14

**Authors:** Ayşen Akkurt Kocaeli

**Affiliations:** 1 Endocrinology and Metabolism, Bursa City Hospital, Bursa, TUR

**Keywords:** body mass index, side effects, body weight loss, obesity, liraglutide

## Abstract

Background

One of the essential chronic diseases is obesity, which negatively affects the individual and society. Liraglutide (LG) is an effective treatment for both obesity treatment and metabolic control. This study aims to show the effect of a 3.0 mg dose of LG, injected subcutaneously once a day, on weight loss and metabolic parameters.

Methods

This retrospective single-center study included 67 patients (60 women and seven men) with a BMI of at least 27 kg/m^2^ with comorbidities or a BMI of at least 30 kg/m^2^. Demographic characteristics, anthropometric measurements, and biochemical data of the participants were evaluated at the end of the four and 16 weeks.

Results

The mean body weight (BW) loss of patients using LG at 16 weeks was -11.71±2.21 kg. After the four and 16 weeks of beginning the LG use, the patients who lost more than 5% of initial BW were 38.8% vs. 76.1%, respectively (p=0.034). The mean baseline Homeostatic Model Assessment-Insulin Resistance Index, hemoglobin A1c, low-density lipoprotein, and triglycerides values were significantly higher than the 4 and 16 weeks (p<0.001). Twenty-two (32.8 %) patients experienced side effects (SE) after starting LG treatment, and the most common SE was found to be nausea (29.4%).

Conclusion

The use of LG, which is not covered by insurance, together with diet and exercise, has been shown to have clinically significant weight loss and a positive effect on glycemic values ​​and lipid profile.

## Introduction

Obesity, which is considered a multifactorial and complex disease that negatively affects health, is one of the most important causes of preventable deaths today. It contributes to the development of many health problems, such as type 2 diabetes mellitus (DM), cardiovascular disease (CVD), hypertension (HT), hyperlipidemia (HL), cerebrovascular disease, various cancers, obstructive sleep apnea syndrome (OSAS), fatty liver, gastroesophageal reflux, polycystic ovary syndrome (PCOS), osteoarthrosis, and depression [1,2]. Therefore, it creates a significant burden on the health budgets of societies. The prevalence of obesity is increasing in our country and reaching epidemic proportions as it is all over the World. In the Turkey Diabetes Epidemiology Studies (TURDEP) conducted in 1998 and 2010, it was seen that the prevalence of obesity in our country increased from 22.3% to 31.2% [3,4]. In 2016, the WHO reported that the country where obesity is most common in Europe is Turkey, with a prevalence of 29.5% [5].

Three methods are used in obesity treatment: lifestyle change, pharmacotherapy, and bariatric surgery. Clinical studies have shown the effectiveness of lifestyle change and behavioral interventions in obesity. Drugs that provide 5% weight loss in three to six months have been accepted as effective in the treatment and have been approved by pharmaceutical institutions worldwide for managing chronic obesity. Adding pharmacotherapy to lifestyle changes helps with further weight loss. It facilitates patients' compliance with treatment and helps improve obesity-related health risks, thus contributing to increased quality of life.

Liraglutide (LG), one of the limited number of obesity drugs in our country, is a long-acting GLP-1 receptor agonist (GLP-1 RA) that is resistant to metabolism by the dipeptidyl peptidase (DPP)-IV enzyme [6]. GLP-1 analogs induce weight loss through many central and peripheral mechanisms. It stimulates glucose-dependent insulin release, reduces the glucagon response, and reduces appetite by slowing gastric emptying [7]. In vitro studies have shown that liraglutide has a central effect and directly stimulates the "cocaine- and amphetamine-regulated transcript" and "pro-opiomelanocortin" neurons and indirect inhibition in neurons expressing "Agouti-related peptide" and "neuropeptide Y" in the arcuate nucleus of the hypothalamus [8]. Thus, appetite is suppressed, energy intake is reduced, and weight loss occurs with these mechanisms. The positive effects of LG on weight loss and metabolic parameters have been emphasized in various studies [9-13].

In Turkey, LG 3 mg (Saxenda®; Novo Nordisk, Bagsvaerd, Denmark) was approved for treating obesity in May 2018. In this study, we aimed to evaluate the effects of LG treatment on weight loss, glycemic and lipid parameters, and the side effects (SE) of this drug as a contribution to the limited number of studies conducted in our country.

## Materials and methods

Study design and participants

This single-center and retrospective study was approved by the Ethics Committee of Bursa City Hospital (approval number 2023-19/2) and was performed per the Declaration of Helsinki. 

The 67 participants between 18 and 65 years old who used liraglutide for at least 16 weeks due to obesity treatment between July 2020 and September 2022 in Bursa City Hospital were included in the study. Individuals who had undergone bariatric surgery, previously used glucagon-like peptide-1 receptor agonists (GLP-1RA) or another drug that affects weight, and had diseases that could cause weight loss, such as cancer, physiatric disease, eating disorders, and chronic kidney disease, were not included in the study. Pregnant or breastfeeding women are also excluded. LG was given to obese people (BMI>30kg/m2) who could not achieve adequate weight loss despite complying with lifestyle changes or to people with BMI>27 kg/m2 and at least one comorbidity (uncontrolled diabetes mellitus (DM), hypertension (HT), obstructive sleep apnea syndrome (OSAS), hyperlipidemia (HL), etc.)

Treatment was started with 0.6 mg daily, and the dose was titrated weekly and increased to 3 mg/day, according to side effects (SEs). All patients were also given a personalized low-calorie-restricted diet and at least 150 minutes of weekly physical activity.

The participants' diagnoses, medications, prescriptions, demographic characteristics, and laboratory results were accessed in the hospital computer database. The body weight (BW), body mass index (BMI), comorbidities of the patients, and follow-up laboratory results at four and 16 weeks were recorded. The patients were questioned about possible drug-related SEs at each follow-up examination.

The BW was detected on a scale without shoes and extra clothing. BMI was calculated as the BW (kilograms) divided by the squared height (in meters). All biochemical parameters were analyzed from serum samples after eight hours of fasting. Plasma values of fasting glucose (FG), fasting insulin (FI), glycosylated hemoglobin (HbA1c), and lipid profile [triglycerides (TG) and low-density lipoprotein (LDL)] were recorded. Homeostatic Model Assessment-Insulin Resistance Index (HOMA-IR): FG (mg/l)' FI (mU/l) /405 was used to measure insulin resistance for all individuals. Type 2 DM, prediabetes, HT, HL, OSAS, PCOS, and a history of CVD were obtained from the patient's records. Prediabetes and type 2 DM were diagnosed according to the American Diabetes Association's diabetes diagnostic criteria [14].

Statistical analysis 

We used the IBM SPSS 21.0 Statistic version 23 package program (IBM Inc., Armonk, New York) and performed statistical analyses. Continuous variables were expressed as mean ± standard deviation for descriptive statistics, and categorical variables were expressed as frequency and percentages. The Shapiro-Wilk test was used to test the normal distribution. The mean values of variables with normal distribution were compared using Student's t-test or analysis of variance (ANOVA) and those without a normal distribution by the Mann-Whitney U test. The significance level was considered as p-values <0.05. 

## Results

Seventy-one patients were evaluated in the study. Due to the higher cost of LG, 10 participants could not continue the treatment for 16 weeks. Therefore, it was not included in the statistical analysis. Sixty (89.5%) women and seven (10.5%) men with a mean age of 42.8 ± 4.4 years met the inclusion criteria. At the beginning of the study in patients, mean BW and BMI were 103.8±18.7 kg and 35.2±7.21kg/m2. The participants' baseline characteristics are presented in Table 1. Patients were classified as overweight, class 1, class 2, and class 3 obese according to their BMI. It was determined that most patients were in the class 2 obese (n=17, 25.3%). Of the study patients, 19 (28.4%) were prediabetic, 45 (67.1%) were normoglycemic, and three (4.5%) were diabetic. There was no concomitant disease in 38 (56.7%) patients. Other comorbidities are shown in Table 1. 

**Table 1:** Characteristics of the participants at baseline BMI - body mass index; CVD - cardiovascular disease; OSAS - obstructive sleep apnea syndrome; PCOS - polycystic ovary syndrome

Variables	(n = 67)
Demographic parameters	
Age, years	42.8 ± 4.4
Gender (female), n (%)	60 (89.5)
Anthropometric parameters	
Weight, kg	103.8 ± 18.7
BMI, kg/m²	35.2 ± 7.21
Overweight (27 to 29.9 kg/m^2^), n (%)	5 (7.5)
Obese class 1 (30 to 34.9 kg/m^2^), n (%)	29 (43.3)
Obese class 2 (35 to 39.9 kg/m^2^), n (%)	17 (25.3)
Obese class 3 (>40 kg/m^2^), n (%)	16(23.9)
Diabetes status	
Normoglycemic, n (%)	45 (67.1)
Prediabetic, n (%)	19 (28.4)
Diabetic, n (%)	3 (4.5)
Hypothyroidism, n (%)	9 (13.4)
Hypertension, n (%)	14 (20.9)
Hyperlipidemia, n (%)	26 (38.8)
CVD	2 (2.9)
OSAS	5 (7.4)
PCOS	7 (10.4)

In this study, all patients reached the LG 3 mg/day target dose and were followed up. The mean BW levels decreased from 103.8±18.7 kg at the beginning of the therapy to 97.6± 17.5 at four weeks and 92.1± 16.4 at 16 weeks. Comparative analyses were found between baseline and four weeks (p=0.023), baseline and 16 weeks (p<0.001), and four and 16 weeks (p=0.019). The mean BMI decreased from 35.2 ± 7.21kg/m2 at baseline to 33.72±7.22 kg/m2 at four weeks and 29.61± 7.14 kg/m2 at 16 weeks. Comparative analyses were conducted between baseline and four weeks (p=0.045), baseline and 16 weeks (p<0.001), and four and 16 weeks (p=0.034). At the end of the 16 weeks, the percentage of body weight loss (BWL) change was found to be comparable between obesity classes 1, 2 and 3 (-9.81±1.93 %, -11.02±2.11 %, and -12.94±2.94respectively; p=0.954), and similar rates of ≥5% BWL were achieved between the three groups (72.6 %, 74.8 % and 78.5 %, respectively; p=0.623).

When evaluated according to their average BWL, the mean BWL and BMI loss of patients using LG in the first four and 16 weeks after treatment initiation were -6.17 ± 1.34 kg, -1.51 ± 1.25 kg/m2, and -11.71±2.21 kg, -5.56±1.88 kg/m2, respectively (Table 2). After the four and 16 weeks of beginning the LG use, the patients who lost more than 5% of initial BW were 38.8% vs. 76.1%, respectively (p=0.034). At four weeks, 14.9% of participants had ≥10% BWL, and this rate increased to 59.7% at 16 weeks (Table 2).

**Table 2:** The weight loss achieved with the use of liraglutide for four and 16 weeks Data are expressed as mean ± SD. Statistically significant values (p<0.05) are shown in bold. BWL - body weight loss; BMI - body mass index

Variables (n=67)	4 weeks	16 weeks	p-value
BWL, kg	-6.17 ± 1.34	-11.71±2.21	<0.001
BWL change %	-4.97± 1.09	-10.94±2.79	<0.001
BWL ≥5% of baseline, n (%)	26 (38.8)	51 (76.1)	0.034
BWL ≥10% of baseline, n (%)	10(14.9)	40(59.7)	0.011
BMI loss, kg/m^2^	-1.51±1.25	-5.56±1.88	<0.001

Changes in metabolic parameters such as fasting glucose (FG), fasting insulin (FI), homeostatic model assessment-insulin resistance (HOMA-IR), glycosylated hemoglobin (HbA1c), low-density lipoprotein (LDL), and triglycerides (TG) values before starting treatment and at the four and 16 weeks are summarized in Table 3. A statistically significant difference was observed between the baseline, week four, and week 16 mean HOMA-IR values (p<0.001). The baseline HOMA-IR levels were statistically significantly higher than week four and week 16 HOMA-IR levels (p<0.001). A statistically significant difference was observed in HbA1c levels between baseline, four weeks, and 16 weeks (p<0.001). The 16-week mean HbA1clevels were statistically significantly lower than the baseline and 4-week mean HbA1c levels (p<0.001). Similar findings were shown for mean FG and FI levels, with the 16-week levels significantly lower than baseline and 4-week (p<0.001, p<0.001, respectively). There was a significant decrease in baseline LDL and TG concentrations at the end of the four and 16 weeks (p<0.001, p<0.001, respectively). In contrast, the difference between the four and 16 weeks was insignificant (p=0.234, p=0.089, respectively).

**Table 3:** Metabolic parameters of participants at baseline and follow-up Data are expressed as mean ± SD; *p - comparisons between baseline and 4 weeks; †p - comparisons between 4 and 16 weeks; §p - comparisons between baseline and 16 weeks; p<0.05 signifies statistically significant values FG - fasting glucose; FI - fasting insulin; HOMA-IR - homeostatic model assessment-insulin resistance; HbA1c - glycosylated hemoglobin; LDL - low-density lipoprotein; TG - triglycerides

Variables n=67	Baseline	4 weeks	16 weeks	p*	p^†^	p^§^
FG (mg/dl)	108.27 ± 29.76	101.18 ± 25.43	97.68 ± 21.27	<0.001	<0.001	<0.001
FI (IU/ml)	24.45 ± 19.78	22.17 ± 16.78	20.45 ± 14.55	<0.001	<0.001	<0.001
HOMA-IR	5.25±3.91	3.98 ± 2.45	3.06 ± 1.97	<0.001	<0.001	<0.001
HbA1c (%)	5.93 ± 0.88	5.85 ± 0.79	5.76 ± 0.71	<0.001	<0.001	<0.001
LDL (mg/dl)	146.9 ± 37.9	129.7 ± 24.5	118.3 ± 21.7	<0.001	0.234	<0.001
TG (mg/dl)	201.2 ± 64.3	187.9 ± 45.7	176.2 ± 39.3	<0.001	0.089	<0.001

While 45 (67.2%) patients did not experience any SEs after starting LG treatment, the most common SEs were nausea (29.4%), abdominal pain (11.8%), vomiting (10.3%), diarrhea (7.2%), and others (15.9%) (headache, dyspepsia, influenza-like symptoms, constipation). Despite these digestive SEs, none of the patients discontinued their treatment.

## Discussion

This study aimed to demonstrate the effectiveness and SE profile of overweight and obese patients with LG treatment evaluated in clinical practice. The findings of this retrospective study showed that mean BW, BMI, FG, FI, HOMA-IR, HbA1c, LDL, and TG levels were significantly reduced in obese or overweight patients at the 16-week follow-up. 

Obesity is a chronic disease associated with high morbidity and mortality risks and limited quality of life that require long-term medical attention. In addition, the increase in health expenditures brings heavy burdens to the country's economies. Treatment options include diet and exercise, medication, or surgery. Many drugs with different mechanisms of action can be used to treat obesity. Although pharmacotherapy is an effective method in the treatment of obesity, drug costs are often a limiting factor. Obesity treatment options worldwide include phentermine, phentermine/topiramate, lorcaserin, naltrexone/bupropion, diethylpropion, orlistat, and LG. In Turkey, only orlistat and LG are approved for use in treating obesity. Pancreatic lipase inhibitor orlistat has serious gastrointestinal SEs, and it is difficult to achieve tolerability. In a European study, GLP-1 analogs are more effective in weight loss than orlistat and glimepirid [15]. In another retrospective study from Spain, BWL with LG (-7.7 kg) was significantly greater than that observed with orlistat (-3.3 kg), and approximately two and a half times more patients lost at least 5% of their initial BW with LG than with orlistat [16]. Opioid antagonists with antidepressant effects Naltrexone-bupropion, sympathomimetic phentermine+ topiramate, and pramlintide were found to be as effective as LG in weight loss, but they were not recommended due to SEs; therefore, the use of LG comes to the fore [17].

The SCALE randomized controlled clinical trial followed 3731 participants with obesity receiving LG; over 13 months, 63.2% and 33.1% of all participants significantly lost at least 5% and 10% of their BW, respectively [9]. In a meta-analysis, Konwar et al. included approximately 6000 patients who did not have DM but were obese and using LG and observed 2.8-11.8 kg of BWL in their follow-up at 12-56 weeks [18]. Recently, Cetiner et al. from Turkey evaluated 201 patients using LG for 12 months and showed significant BWL. After three months from the LG treatment, 72.14% of the patients (n=145/201), and at the end of six months, almost all (n=96/106) had more than 5% weight loss observed. Additionally, the mean weight loss was 17.79 ± 8.93 kg for those who continued treatment for 12 months [19]. Our investigation determined that LG 3.0 mg for patients with obesity or overweight led to significant BWL of 6.1 to 11.7 kg (4.9%-10.9%) at four and 16 weeks of treatment, respectively. The results revealed that over 75 % and 55% of the participants who used the LG for the initial 16 weeks achieved ≥ 5% and ≥10%BWL, respectively. These findings are similar to or mostly higher than those reported in previous randomized controlled clinical trials [9, 20-22]. Additionally, our results are superior to those of Italian [10], Canadian [11], and Spanish [16]; real-life studies demonstrated that 64% to 68% of patients exhibited >5% BWL and 20% to 35% of patients exhibited >10% BWL at four to seven months of treatment. In contrast, our study compared to a smaller cohort from Switzerland (n=54) showed that the percentage of patients reaching ≥5% weight loss at 16 weeks was lower [12]. In that study with a four-month follow-up, 87% of subjects showed ≥5% BWL, and this percentage increased to 96% at 10 months [12]. This difference in results can be attributed to different nutritional habits between populations. In Turkey, where dietary habits consume extremely high amounts of carbohydrates, transitioning to a low-calorie diet combined with the appetite-suppressing effects of LG may have resulted in more significant weight loss in a short period.

In patients using LG, diabetes regulation is impaired due to its effect on decreased appetite and gastrointestinal intolerance. In addition, significant weight losses were noted. A recently published randomized placebo-controlled trial of LG found that patients who experienced nausea achieved a more significant absolute weight loss [23]. Previous studies found a direct correlation between drug dose and weight loss [10,20]. The BWL increases, especially when the dose is increased to 2.4-3.0 mg/day [24]. In this study, all patients started with LG 0.6 mg and reached the 3mg maximum dose with dose titration within four weeks. Therefore, the relationship between weight loss and drug dose could not be evaluated.

In another study conducted in Canada, the effectiveness of LG was determined according to the degree of obesity, and no difference in the effectiveness of LG was found in stage 1, stage 2, and stage 3 obese patients [11]. In our study, the percentage of BW change and ≥5% BWL were similar between obesity classes. 

Our study, consistent with a recent meta-analysis, showed a significant decrease in glycemic control variables such as HbA1c, FG, FI, HOMA-IR, and fasting lipid parameters [25]. However, it is unclear whether GLP-1RAs ameliorate metabolic parameters to the same extent in obese patients with and without diabetes. Santini et al. found considerable improvement in triglycerides, glucose profile, and insulin resistance but no significant changes in total cholesterol, LDL, or high-density lipoprotein (HDL) levels [12]. 

The SEs of LG were consistent with findings in previous reports. Our evaluation of LG's SEs showed that 67.1% did not experience any SEs, while 32.9% reported SEs. The most common SE was nausea, observed in approximately one in three participants. Other reported SEs included abdominal pain, vomiting, diarrhea, headache, dyspepsia, influenza-like symptoms, and constipation. However, these SEs are mild to moderate and transient with symptomatic treatment or dose reduction [26]. In our study, patients had nausea during the increase in dose, especially in the transition to 1.2-1.8 mg/day, and many of them were given only symptomatic treatment. Also, LG is not recommended for those with a personal or family history of pancreatitis and multiple endocrine neoplasia (MEN) 2A and 2B [27].

Despite the positive effectiveness in metabolic control and weight loss of LG, it continues to be an expensive treatment in our country. We think that GLP-1 analogs should be included in the scope of health insurance, considering that they will provide severe benefits in the fight against obesity in the future.

This study has several limitations. The first is that the study was single-center, retrospective, and small sample size. Secondly, it cannot provide long-term BW changes because the follow-up lasted only 16 weeks. Due to the cost of the medicine, the duration of use of patients is shortened, and long-term results cannot be evaluated. Finally, most of our study participants were women; thus, studies with a greater number of both genders are needed.

## Conclusions

In recent years, medical treatments for obesity, in addition to lifelong adequate and balanced nutrition, physical activity, and behavioral therapies, have come forth. This study showed a clinically significant decrease in BW and improved cardiometabolic parameters in four and 16 weeks of treatment with LG. It stands as a safe and effective medical treatment modality for addressing the issue of obesity. Unfortunately, financial limitations in drug use remain a significant obstacle.
